# Survival after acute colon diverticulitis treated in hospital

**DOI:** 10.1007/s00384-014-1946-3

**Published:** 2014-07-03

**Authors:** Tom-Harald Edna, Aras Jamal Talabani, Stian Lydersen, Birger Henning Endreseth

**Affiliations:** 1 Department of Surgery, Levanger Hospital, North-Trondelag Hospital Trust, Levanger, Norway; 2 Unit for Applied Clinical Research, Faculty of Medicine, Institute of Cancer Research and Molecular Medicine, Norwegian University of Science and Technology, Trondheim, Norway; 3 Regional Centre for Child and Youth Mental Health and Child Welfare – Central Norway, Faculty of Medicine, Norwegian University of Science and Technology, Trondheim, Norway; 4 Department of Gastroenterological Surgery, St Olavs Hospital, University of Trondheim, Trondheim, Norway; 5 Institute of Cancer Research and Molecular Medicine, Faculty of Medicine, Norwegian University of Science and Technology, Trondheim, Norway

**Keywords:** Acute colonic diverticulitis, Short-term survival, Long-term survival

## Abstract

**Purpose:**

The aim of this study was to determine the short- and long-term relative survival as well as the causes of death in patients treated in hospital for acute colonic diverticulitis.

**Materials and methods:**

The study included all patients treated at Levanger Hospital for acute colonic diverticulitis between 1988 and 2012. Vital statistics were complete. The median observation time was 6.95 years (range 0.28–24.66) or until death.

**Results:**

In total, 650 different patients were hospitalized with acute colonic diverticulitis. Among these patients, there were 851 admissions for the same disease during the 25 years. The admissions had the following diagnoses: simple diverticulitis, 738; abscess formation , 44; perforation and purulent peritonitis, 47; perforation and fecal peritonitis, 9; and intestinal obstruction, 13. During the observation time, 219 were dead and 431 were still alive. After the first admission, the 100 day relative survival in patients with uncomplicated diverticulitis was 97 % (CI 95 to 99), with abscess formation 79 % (62 to 89), with purulent peritonitis 84 % (69 to 92), with fecal peritonitis 44 % (10 to 74), and with intestinal obstruction 80 % (38 to 96). After surviving the first 100 days, the estimated 5-year relative survival in the remaining 609 patients was 96 % (CI 92 to 100) and 10-year survival was 91 % (CI 84 to 97). In patients who survived the first 100 days, the different subtypes of diverticulitis yielded no significant differences in long-term relative survival. All patients who had been admitted with ASA score 4 were dead after 2 years.

## Introduction

Acute colonic diverticulitis is an increasingly common acute abdominal condition in the Western world. Most patients have uncomplicated acute diverticulitis, while 9–35 % develop serious acute complications due to colonic perforation, with abscess formation or diffuse peritonitis, or intestinal obstruction [1–5].

Inhospital mortality has been shown to occur in 0 to 17 % of patients with abscess formation and 0.4 to 45 % of patients with perforation and generalized peritonitis [6–11]. In series that also include patients with uncomplicated diverticulitis, the inhospital mortality rate is between 0.5 and 7 % [6, 7].

Population-based estimates of short- and long-term survival after admission for the different types of acute colonic diverticulitis are scarce. Patients with perforation or abscess had 15.5 % 1-year mortality in a recent study from the UK [12]. Long-term mortality after different types of diverticulitis has been studied by Binda et al. [13] and after perforated diverticulitis by Vermeulen [14]. In those who survived the first episode with diverticulitis, long-term survival was not associated with the severity of the primary disease.

The objective of the present study was to assess short- and long-term relative survival in patients treated for the different subtypes of acute colonic diverticulitis in hospital. We also studied the causes of death in all patients.

## Patients and methods

### Study population

All patients hospitalized because of acute diverticulitis at Levanger Hospital, between January 1988 and December 2012, were identified through the patient administrative system. The records were reviewed and clinical data collected. A total of 650 patients were admitted 851 times: 519 were admitted once, 91 twice, 25 three times, 9 four times, 1 five times, 4 six times, and 1 ten times.

We classified the cases as uncomplicated or complicated acute diverticulitis. Acute complicated diverticulitis was further classified into four subcategories: abscess formation (Hinchey stage I or II), bowel obstruction related to stenosis, perforation with purulent peritonitis (Hinchey stage III), or fecal peritonitis (Hinchey stage IV) [15].

The physical status at admission was categorized into five groups using the American Society of Anesthesiologists ASA score [16].

Survival time was defined as time from first admission with acute colonic diverticulitis until death, regardless of cause. Patients who were alive at the end of April 2013 counted as cencored cases. The cause of death was available in the records of 613 patients, and for the remaining 37 patients, cause of death was obtained from the Cause of Death Registry, Statistics Norway.

### Major endpoints, short- and long-term relative survival

The primary study endpoint was death. Short-term relative survival was reported as 100-day relative survival. Long-term relative survival was estimated for the patients who had survived the first 100 days to see if the patients who survived the acute episode of acute diverticulitis still had excess mortality compared to the general population. The long-term survival was calculated from the first admission for acute diverticulitis.

### Statistical methods

Relative survival aims to measure mortality in excess of what would be expected for the study population if it did not have acute diverticulitis. In relative survival analysis, the observed survival in the group with diverticulitis is divided by the expected survival of a comparable group in the general Norwegian population with respect to age, sex, and calendar year of investigation. Using relative survival, consideration was made for the change in the age and gender in the population during the 25 years of study, and figures for excess mortality in the study population were given directly. Relative survival was estimated using the Ederer II method and analyzed with STATA 12 (Stata Corp LP, College Station, TX, USA) [17, 18]. Multivariable analyses were done using a full likelihood approach. Norwegian population survival probabilities for every year from 1988, by sex and age, were downloaded from the Human Mortality Database [19].

Two-sided *p* values <0.05 were considered significant. Ninety-five percent confidence intervals (CIs) are reported where relevant. Medians are reported with range (minimum to maximum) where relevant.

## Results

### Study population and primary treatment

Baseline figures for the subcategories of diverticulitis in the 650 patients with 851 different admissions are given in Table 1. Diverticulitis with perforation leading to purulent or fecal peritonitis occurred in 56 patients. Eighty-nine percent (50/56) of the perforations occurred during the patients’ first admission. Among the 552 patients with uncomplicated diverticulitis at the first admission, 123 (22.3 %) were later readmitted, mostly with uncomplicated disease. After complicated diverticulitis, readmissions were less frequent. Patients with abscess formation had the longest time interval between onset of symptom until admission to hospital, 7 days (1–35).

**Table 1:** Baseline characteristics in relation to subcategory of acute diverticulitis. Medians and ranges are given

	Subcategory of acute diverticulitis during the first admissions in 650 patients				
	Uncomplicated	Perforated with abscess	Perforated with purulent peritonitis	Perforated with fecal peritonitis	With acute intestinal obstruction
	(*n* = 552)	(*n* = 38)	(*n* = 42)	(*n* = 8)	(*n* = 10)
Age	64 (24–98)	69 (43–93)	66 (27–93)	76 (31–91)	78 (69–94)
Male sex (%)	229 (41.5)	14 (36.8)	17 (40.5)	2 (25.0)	3 (30.0)
Time interval^a^	2 (0–30)	7 (1–21)	2 (1–30)	1 (1–5)	4 (1–14)
CRP					
At admission	91 (1–452)	150 (33–366)	231 (0–620)	6 (5–299)	96 (8–271)
Highest	114 (1–453)	223 (33–524)	301 (26–620)	273 (5–417)	119 (59–363)
Hospital stay in days	3 (0–30)	7 (1–35)	10 (0–73)	13 (1–31)	14 (7–22)
Patients readmitted after first admissions					
No	429	34	39	8	9
Yes	123	4	3	0	1
No. of readmissions	191	5	4	0	1
Subcategory of diverticulitis during readmissions					
Uncomplicated	178	4	3	0	1
Abscess	5	1			
Purulent peritonitis	5				
Fecal peritonitis	1				
With acute obstruction	2		1		

^a^Days from first symptom to admission

A surgical procedure was performed in 88/650 patients (13.5 %). A total of 80 were operated at their first admission, 4 at the second admission, 2 at their third admission, 1 at the forth admission, and 1 at the tenth admission. Hartmann procedure was performed in 54 patients, exploratory laparotomy in 13, resection with primary anastomosis and diverting stoma in 9, primary anastomosis without stoma in 7, peritoneal lavage and drainage in 2, laparotomi with drainage of abscess in 2, and only proximal stoma in 1. Radiological abscess drainage was done in nine patients. Antibiotics had been given to all patients with complicated diverticulitis and in 726/738 admissions (98 %) with uncomplicated diverticulitis.

During the observation time, 219 patients were dead and 431 were still alive. The median observation time was 6.95 years (0.28–24.66) or until death.

### Short-term survival

**Fig. 1:**
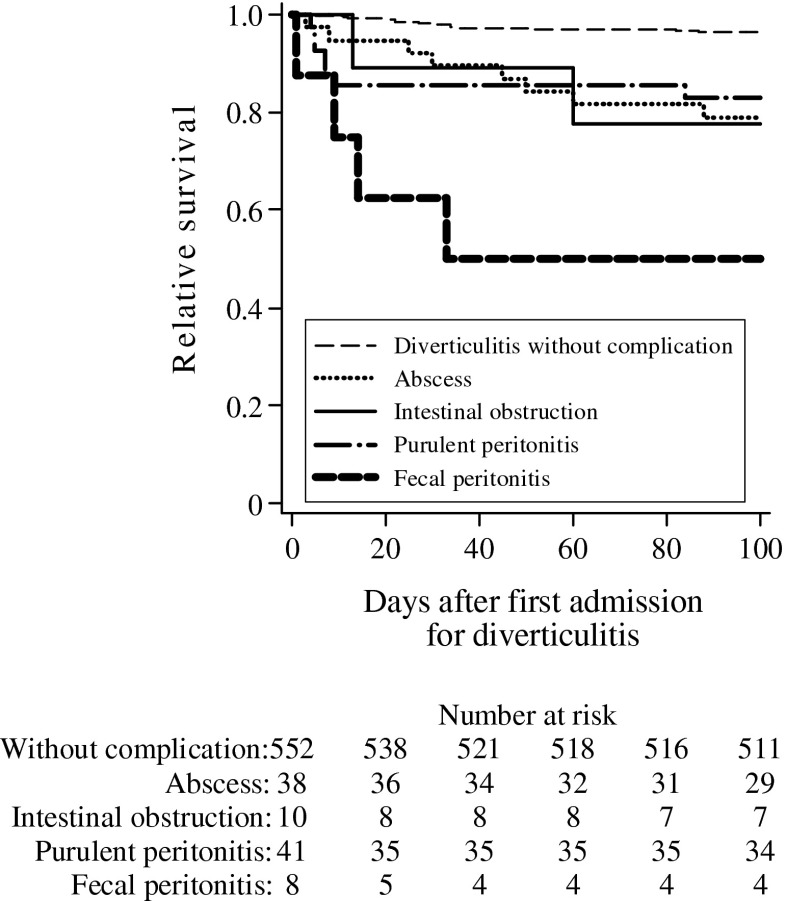
Relative survival 100 days after first episode of acute colonic diverticulitis

The total 100-day mortality rate was 7.5 % (49/650). Forty-one patients died during their first admission. Six patients died after a second admission and two after a third admission because of acute diverticulitis during this time interval. The 100-day relative survival curves in relation to the subcategories of acute diverticulitis are shown in Fig. 1.

Table 2 shows the results of unadjusted and adjusted analysis of relative survival during the first 100 days after the first admission for acute colonic diverticulitis. In the multivariable analysis, higher ages and high ASA scores, abscess formation, and perforation with fecal peritonitis were significantly associated with a shorter 100-day relative survival. Perforation with fecal peritonitis was the most serious form of the disease with high mortality and with an adjusted hazard ratio of 10.5 (CI 3.12 to 35.1) compared to uncomplicated acute colonic diverticulitis.

**Table 2:** Multivariable analysis of 100 days relative survival after first admission for acute colonic diverticulitis

	No. (%) *N* = 650	Unadjusted hazard ratio	*p* value	100-day relative survival	Adjusted^a^ hazard ratio	*p* value
Gender						
Male	265 (40.8)	1		95 % (92 to 98)	1	
Female	385 (59.2)	1.30 (0.68 to 2.51)	0.43	94 % (91 to 94)	0.71 (0.35 to 1.46)	0.35
Age						
< 65 years	321 (49.4)	1		100 % (98 to 100)	1	
65–79 years	211 (32.5)	32.0 (4.2 to 242)	0.001	91 % (86 to 94)	15.9 (2.03 to 123)	0.008
80 + years	118 (18.2)	60.4(7.9 to 458)	<0.001	86 % (78 to 92)	15.9 (1.97 to 129)	0.010
ASA score						
1–2	461 (70.9)	1		99 % (98 to 100)	1	
3	163 (25.1)	12.1 (4.8 to 31.0)	<0.001	90 % (85 to 94)	4.5 (1.7 to 11.7)	<0.001
4	26 (3.9)	123 (47.1 to 320)	<0.001	39 % (22 to 55)	37.1 (11.7 to 118)	<0.001
Type of diverticulitis						
Uncomplicated	552 (8.9)	1		97 % (95 to 99)	1	
Abscess formation	38 (5.8)	7.0 (3.05 to 16.1)	<0.001	79 % (62 to 89)	3.26 (1.34 to 7.93)	0.009
Perforation with purulent peritonitis	42 (6.5)	5.4 (2.3 to 12.9)	<0.001	84 % (69 to 92)	1.23 (0.42 to 3.53)	0.71
Fecal peritonitis	8 (1.2)	30.4 (10.3 to 90)	<0.001	44 % (10 to 74)	10.5 (3.12 to 35.1)	<0.001
With intestinal obstruction	10 (1.5)	7.1 (1.6 to 30.9)	0.009	80 % (38 to 96)	1.26 (0.24 to 6.51)	0.78

^a^Adjusted for all factors

### Diagnosis specific short-term mortality

Table 3 shows the main causes of a short-term lethal outcome. In patients with perforation, the main cause of death was septic complications (11/12). Ten out of 19 (53 %) patients with uncomplicated diverticulitis died of cardiovascular disease.

**Table 3:** Causes of death during the first 100 days after the first admission for acute colonic diverticulitis, in relation to type of diverticulitis in 1988–2012

Main cause of death	Type of acute diverticulitis				
	Uncomplicated diverticulitis (*n* = 552)	With abscess (*n* = 38)	Perforated with purulent peritonitis (*n* = 42)	Perforated with fecal peritonitis (*n* = 8)	With acute intestinal obstruction (*n* = 10)
Cardiovascular disease	10	2			
Cancer	2	2		1	
Sepsis	2	4	8	3	1
Other	5^a^				1^b^
Total	19/552 (3.4 %)	8/38 (21 %)	8/42 (19 %)	4/8 (50 %)	2/10 (20 %)

^a^One each of fulminant hepatitis, uremia, rheumatoid arthritis with multi organ failure, Wernicke’s encephalopathy, and one unknown

^b^Femoral neck fracture

### Long-term survival

**Fig. 2:**
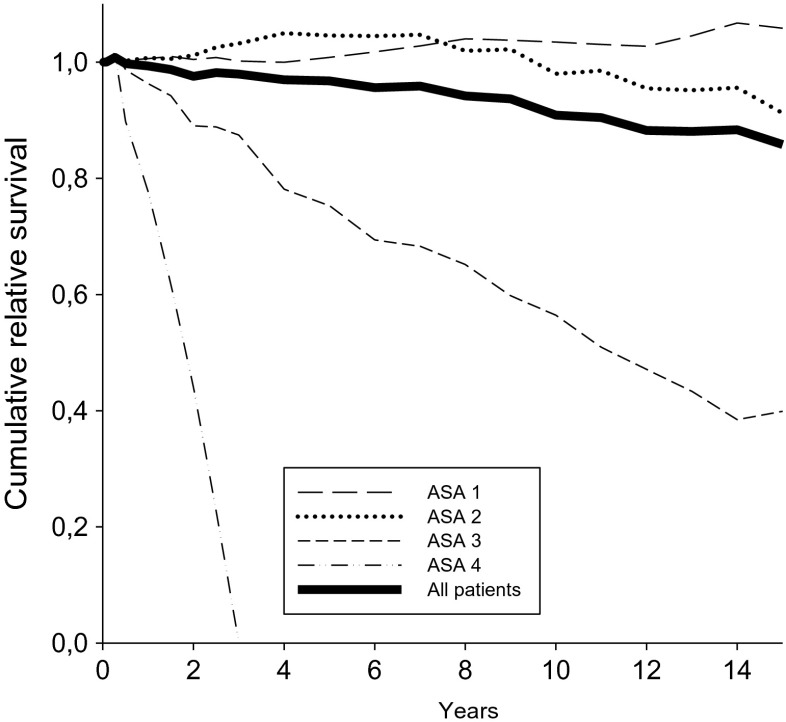
Long-term relative survival in patients having survived beyond 100 days after first admission for acute diverticulitis, in relation to ASA score

Six hundred and nine patients survived the first 100 days after the first admission. In this group, the estimated relative survival decreased slightly by the years and was 96 % (CI 92 to 100) after 5 years, 91 % (CI 84 to 97) after 10 years, and 86 % (CI 76 to 95) after 15 years. The corresponding figures after uncomplicated diverticulitis were 97 % (CI 92 to 100) after 5 years, 91 % (CI 84 to 98) after 10 years, and 87 % (CI 76 to 97) after 15 years. After diverticulitis with abscess formation or free perforation (Hinchey I–IV), it was was 91 % (CI 75 to 100) after 5 years, 85 % (CI 62 to 103) after 10 years, and 69 % (CI 40 to 95) after 15 years. Figure 2 shows the estimated relative long-term survival in all patients in relation to ASA score at admission. All patients with ASA score 4 died within 3 years. Having survived the first 100 days, only one patient with acute diverticulitis later died directly after a new episode of acute diverticulitis. This episode was complicated with perforation, peritonitis, and sepsis.

Table 4 shows the results of unadjusted and adjusted analyses of different factors in relation to long-term relative survival in patients who survived the first 100 days after the first admission for acute colonic diverticulitis. The long-term survival beyond 100 days was unrelated to the different subtypes of acute diverticulitis, while higher ages, male gender, and higher ASA scores were associated with worse survival.

**Table 4:** Multivariable analysis of long term relative survival in patients who survived the first 100 days after the first admission for acute colonic diverticulitis

	No. (%) *N* = 609	Unadjusted hazard ratio	*p* value	5-year relative survival	Adjusted^a^ hazard ratio	*p* value
Gender						
Male	251 (41.2)	1		95 % (88 to 100)	1	
Female	358 (58.8)	0.93 (0.63 to 1.37)	0.70	98 % (92 to 102)	0.66 (0.46 to 0.95)	0.023
Age						
< 65 years	319 (52.4)	1		99 % (95 to101)	1	
65–79 years	192 (31.5)	4.43 (2.75 to 7.15)	<0.001	97 % (89 to 103)	4.22 (2.58 to 6.89)	<0.001
80 + years	98 (16.1)	15.5 (9.59 to 25.2)	<0.001	86 % (64 to 107)	9.80 (5.65 to 17.0)	<0.001
ASA-score						
1–2	455 (74.7)	1		104 % (100 to 107)	1	
3	145 (23.8)	5.90 (4.03 to 8.64)	<0.001	76 % (63 to 87)	2.51 (1.69 to 3.72)	<0.001
4	9 (1.5)	30.9 (13.0 to 73.3)	<0.001	0 % (0 to 0)	7.74 (2.92 to 20.5)	<0.001
Type of diverticulitis						
Uncomplicated	533 (87.5)	1		97 % (92 to 100)	1	
Abscess formation	29 (4.8)	1.22 (0.50 to 2.98)	0.67	98 % (70 to 112)	1.11 (0.51 to 2.40)	0.79
Perforation with purulent peritonitis	35 (5.8)	1.26 (0.62 to 2.58)	0.52	85 % (65 to 97)	1.26 (0.62 to 2.56)	0.52
Fecal peritonitis	4 (0.7)	2.4 × 10^−6^ (0)	0.99	107 % (107 to 107)	3.0 × 10^−6^	0.99
With intestinal obstruction	8 (1.3)	2.28 (0.71 to 7.28)	0.16	137 % (53 to 156)	1.21 (0.39 to 3.72)	0.74

^a^Adjusted for all factors

### Diagnosis specific long-term mortality

Table 5 shows the main causes of death later than 100 days after the first admission of acute colonic diverticulitis. This is compared with the distribution of the main causes of death in North Trondelag county during 1988–2011. The proportion of patients who died from cardiovascular diseases, cancer, or bacterial infections was quite similar, while death caused by chronic obstructive pulmonary disease was more frequent in patients who had had diverticulitis.

**Table 5:** Distribution of causes of death of 178 patients who died after the first 100 days after the first admission for acute colonic diverticulitis, compared with the causes of death in North Trondelag county during 1988–2011

Main cause of death	Patient dead more than 100 days after acute diverticulitis	Total deaths in North Trondelag county
	*n* = 178	*n* = 29,449
Cardiovascular disease	75 (42 %, 33 to 53)	13,037 (44.3 %, 43.5 to 45.0)
Cancer, all forms	35 (20 %, 14 to 27)	6,681 (22.7 %, 22.1 to 23.2)
Bacterial infections, including pneumonia	17 (10 %, 5.6 to 15)	1,734 (5.9 %, 5.6 to 6.2)
Chronic obstructive pulmonary disease	18 (10 %, 6.0 to 16)	1,239 (4.2 %, 4.0 to 4.4)
Other	26 (15 %, 9.5 to 21)	6,758 (22.9 %, 22.4 to 23.5)
Unknown	7 (4 %, 1.6 to 8.1)	

Poisson exact 95 % confidence intervals

## Discussion

The present study confirmed high short-term mortality after perforated diverticulitis. In the group of patients who survived the first 100 days, the long-term survival was the same irrespective of type of diverticulitis and was only slightly curtailed compared to the Norwegian general population. During the entire study period, the patients were admitted from a defined geographical area that is served by one primary hospital which treats all categories of acute diverticulitis. Though the results may not be directly comparable with results from large referral centers because some patients with complicated diverticulitis might be referred to larger centers, it is worth considering that some patients with a grave prognosis may not be transportable and may die at the local hospital. Nonetheless, the present hospital most likely had a larger proportion of patients with uncomplicated disease compared to a center serving as a referral hospital.

The age distribution and death rates in the general population have changed during 25 years. In using relative survival analysis instead of overall survival analysis, we adjusted for these changes during the study period.

### Short-term survival

Short-term survival is commonly reported as inhospital mortality or 30-day mortality. The present report chose 100-day mortality to denote short-term survival whether the patient died in hospital or not. Although most complications after a serious infection or an operation are evident within the first 4 weeks after an operation, death frequently occurs much later because of efficient intensive care units. Therefore, The Accordion Severity Grading System of surgical complications has proposed that the time horizon for postoperative deaths should be extended to 100 days [20]. Thus, in the present study, the short-term relative survival was defined as survival 100 days after the first admission due to an acute colonic diverticulitis and included patients who had been operated and those who had not operated.

Mortality in patients with uncomplicated diverticulitis was infrequently caused directly by this infection itself. The main causes were associated diseases, formost cardiovascular diseases, and an end stage of other serious diseases. Acute perforation with fecal peritonitis was the most serious complication, with a higher mortality rate than in cases with acute perforation and purulent peritonitis. In the multivariable analysis, patients with purulent peritonitis did not have a significantly reduced survival. This was probably a type II error. Haglund et al. in 1979 reported 24 % mortality in 42 patients with diffuse peritonitis and 45 % mortality in 7 patients with diffuse fecal peritonitis [21]. This was in accordance with findings in the present study as well as those of a recent Dutch study [14] that reported inhospital mortality in patients operated for all types of perforation as 26 %, highest in fecal peritonitis with 48 %. A British population-based study of patients with perforated diverticulitis found no mortality in Hinchey grade I, 17 % in Hinchey grade II, 23 % in Hinchey grade III, and 45 % in Hinchey grade IV [11].

A study from a Finnish referral center reported an overall inhospital mortality of 2.3 %, being 1 % in uncomplicated diverticulitis and only 5.5 % among pasients admitted for diverticular perforations [4]. The low mortality may be related to selection bias compared to the present study. A nationwide Canadian study of prognosis in diverticulitis reported an inhospital mortality rate of 0.4 % in the ages 51–60 years and 3.3 % above 60 years [6]. In some patients, death in the course of acute colonic diverticulitis was the end stage of another fatal disease, like cancer. In uncomplicated diverticulitis, cardiovascular disease was the main cause of death within 100 days. In the patients with a fatal short-term outcome after complicated diverticultis, septic complications predominated.

### Long-term survival

The estimated long-term relative survival in patients who survived the first 100 days after acute diverticulitis was unrelated to the subtype of diverticulitis. All had a slightly reduced estimated relative survival, and this was related to accompanying diseases, as measured by the ASA score. Only one patient later died because of diverticulitis, this time with perforation. The rest died from other diseases. Similar findings were reported in a recent study [14].

The distribution of causes of death later than 100 days after the first admission in the study population was quite similar to the population of North Trondelag county during the same period.

One exception was chronic obstructive pulmonary disease, which was more prevalent in patients who had been hospitalized because of acute colonic diverticulitis. The relationship has not been reported before and may be of little importance. It could be related to a common pathogenic factor, like smoking [22] or soft tissue weakening, in both diverticulosis and chronic obstructive pulmonary disease.

Nearly all perforations happened during the first stay, and almost all deaths directly related to diverticulitis occurred during the first admission. The present study, therefore, supports the view held by others that elective resection for diverticulitis is not indicated when the intent is to reduce mortality caused by future episodes of diverticulitis. However, it may be considered if the goal is to improve the quality of life in able-bodied patients with continued severe symptoms, including stenosis or fistula [4, 9, 13, 21, 23, 24].

### Weaknesses and limitations of the study

The study was retrospective in nature. We did not base the study only upon diagnosis lists, but controlled every patient’s record for evidence of acute colonic diverticulitis. Nevertheless, the diagnosis would be even more reliable in a prospective study, which requires a set of diagnostic tests to confirm or disprove the diagnosis of acute colonic diverticulitis. The study included only patients treated in hospital. The findings might not be valid for patients with mild disease treated at home.

### Strengths

The hospital served the whole population within a geographical area and treated the whole spectrum of hospitalized patients with acute colonic diverticulitis, including all complications. The endpoint, death, was a very robust parameter and known in all patients. We used relative survival analysis, instead of overall survival analysis. This made it possible to compare survival in the patients directly with survival in the general population and over as long period as 25 years.

## Conclusions

The 100 days’ relative survival was significantly reduced in perforated disease, particularly after fecal perforation. Even patients with uncomplicated diverticulitis had a small reduction of 100 days’ survival, which was mainly due to accompanying diseases. In the patients who survived the first 100 days, the long-term survival was only slightly reduced. It was independent of the type of diverticulitis, but dependent on accompanying diseases.
